# Molecular Analysis of Chinese *Celastrus* and *Tripterygium* and Implications in Medicinal and Pharmacological Studies

**DOI:** 10.1371/journal.pone.0169973

**Published:** 2017-01-12

**Authors:** Xian-Yun Mu, Liang-Cheng Zhao, Zhi-Xiang Zhang

**Affiliations:** Laboratory of Systematic Evolution and Biogeography of Woody Plants, College of Nature Conservation, Beijing Forestry University, Beijing, PR China; Chinese Academy of Medical Sciences and Peking Union Medical College, CHINA

## Abstract

*Celastrus* and *Tripterygium* species, which are used in traditional Chinese medicine, have attracted much attention due to their anti-tumor promoting and neuroprotective activities, in addition to their applications in autoimmune disorders. However, systematic relationships between them and among species are unclear, and it may disturb their further medicinal utilization. In the present study, the molecular analysis of combined chloroplast and nuclear markers of all Chinese *Celastrus* and *Tripterygium* was performed, and clear inter- and intra-genus relationships were presented. The result suggests that *Tripterygium* constitute a natural monophyletic clade within *Celastrus* with strong support value. Fruit and seed type are better than inflorescence in subgeneric classification. Chinese *Celastrus* are classified for three sections: Sect. *Sempervirentes* (Maxim.) CY Cheng & TC Kao, Sect. *Lunatus* XY Mu & ZX Zhang, sect. nov., and Sect. *Ellipticus* XY Mu & ZX Zhang, sect. nov. The phylogenetic data was consistent with their chemical components reported previously. Owing to the close relationship, several evergreen *Celastrus* species are recommended for chemical and pharmacological studies. Our results also provide reference for molecular identification of Chinese *Celastrus* and *Tripterygium*.

## Introduction

Plants of the genus *Celastrus* and *Tripterygium* (Celastraceae), which have a long history of use in traditional Chinese medicine (TCM), have attracted much interest due to diverse pharmacological activities such as anti-tumor promoting and neuroprotective [1–5]. Celastrol, a pentacyclic triterpene extracted from *T*. *wilfordii*, is found to be a powerful leptin sensitizer, which has potential as an anti-obesity therapeutic agent [6]. Related plant materials are also used as important folk medicines to treat fever, chills, joint pain, and a variety of autoimmune and inflammatory-related conditions [7–8]. *Celastrus angulatus* Maxim. and *T*. *wilfordii* Hook f. have been the main focus of chemical and pharmacological studies among the two genera, which are known as Ku Pi Teng and Lei Gong Teng, respectively. Although hundreds of secondary metabolites including sesquiterpenes, alkaloids, triterpenes, diterpines, and flavonoids have been identified, newer constituents offering more far-reaching, interesting, and applicable prospects are reported recently [9–14].

Comparing to fast developments in chemical and pharmacological studies from a limited number of species, morphology-based subgeneric classification and phylogenetic relationship of these two important TCM lianas is obscure. Although they are morphologically different, two species recognized and composed of *Tripterygium* are nested as a monophyletic clade in *Celastrus* with a moderate support value [15]. Considering the obvious differences in flower and fruit characteristics between these two genera, it is necessary to elucidate their phylogenetic relationship by comprehensive molecular analysis. Despite three sections classified for Chinese *Celastrus* mainly based on inflorescence [16], such scheme is not supported by molecular phylogenetic result, and the relationship of these species and the sections need further investigation. In the previous study, cauline cyme and lunate seeds are found to be distinct characters to one of the maximal supported monophyletic clade in *Celastrus*, Ser. *Laterales* Di [17]. *Celastrus yuloensis*, a new species reported recently [18], is morphologically similar to species in Ser. *Laterales*. Although possesses lunate seeds, *C*. *yuloensis* have completely different inflorescence type, the axillary long cymes. The phylogenetic position of *C*. *yuloensis* will provide useful value for morphology-based subgeneric classification of *Celastrus* in China.

Although exact species identification is fundamental in pharmacological and chemical study, misidentification of rural material may occur occasionally. Because of variable morphology and polymorphic traits inter- and intra-generic of *Celastrus* and *Tripterygium*, specimens are frequently misidentified in herbaria. It is important to identify plant species correctly when new chemical constituents are found from related material, especially in poisoning and mortality cases from a forensic view. Molecular identification brings a good opportunity for accurate identification of herbal medicinal materials [19]. Related method and result are very important for species identification from pharmacological, chemical and forensic perspective in *Celastrus* and *Tripterygium*.

Secondary metabolites are restricted to particular taxonomic groups such as genus, family, or a closely related group [20]. The family Celastraceae is well-known to produce various terpenoid derivatives. Considering that a large number of similar chemistry constituent are found in *C*. *angulatus*, *C*. *orbiculatus*, *C*. *paniculatus*, *C*. *hindsii* and *Tripterygium* [7–8], related constituent may be also existed in closely related species. Due to poor sampling of *Celastrus* species and the limited number of markers used, a more comprehensive analysis of all Chinese *Celastrus* species is necessary [21], which will broaden the utilization of potentially important medicinal germplasm resources, and further validate and refine forensic application of the two genera.

The purpose of this study was to investigate inter- and intra-generic relationships between *Celastrus* and *Tripterygium* in China and to further clarify this relationship, and to evaluate the potential value of inflorescence vs. fruit and seed for subgeneric classification in *Celastrus*. Molecular phylogenetic analysis was performed on all species of *Celastrus* and *Tripterygium* in China with six markers from nuclear and plastid DNA sequences. The results reported pave a way for molecular identification of *Celastrus* and *Tripterygium* and the exploration and utilization of additional species for chemical and pharmacological studies.

## Materials and Methods

### Ethics statement

The field investigation and samples collection in National Nature Reserves were permitted by related management bureaus from Guangxi Nonggang and Hubei Shennongjia. Individuals from Taiwan were sent by Herbarium of Taiwan Forestry Research Institute (TAIF). No specific permissions were required for other locations which are neither privately owned nor protected and the field study did not involve endangered or protected species.

### Plant material

All 20 recognized species of *Celastrus* from China [22], including two newly identified species, *C*. *obovatifolius* and *C*. *yuloensis* [18, 23], and two *Tripterygium* species [21], were included in this study. For the purpose of avoiding potential hybrids that may complicate the data analysis, individuals of these species were selected from their typical distribution area in China. Three species of *Euonymus* L. and one species of *Glyptopetalum* Thwaites were chosen as outgroups based on Mu et al. [15]. Considering the consistency of the samples submitted to GenBank, sequences of two synonyms, *C*. *oblanceifolius* and *C*. *glaucophyllus*, were used to represent *C*. *aculeatus* and *C*. *hookeri*, respectively. A list of sample information and GenBank accession numbers is provided (S1 Table).

### DNA extraction, amplification and sequencing

Two nuclear (ETS and ITS) DNA and four plastid DNA (*psbA-trnH*, *rbcL*, *rpl16*, and *trnL-F*) were employed. Sequences of all samples of *rbcL* marker and some new samples of other species were newly amplified and sequenced. The following primers were used for both amplification and sequencing: 18S-IGS and ETS1F for the ETS region [24]; ITS4 and ITS5 for the ITS region [25]; *psbA*-F and *trnH*-R for the *psbA-trnH* region [26]; 1F and 1024R for the *rbcL* region [27]; F71 and R1516 for the *rpl16* region [28]; and c and f for the *trnL-F* region [29].

Total genomic DNA was extracted from fresh silica gel-dried leaves using a plant genomic DNA kit (Tiangen Biotech CO., LTD, Beijing, China) following the manufacturer’s instructions. Polymerase chain reaction (PCR) amplification was performed in a final volume of 20 μL with ddH_2_O (14.1 μL), Taq buffer (2 μL), dNTPs (1.6 μL), primers (forward and reverse, 0.5 μL per primer), Taq-polymerase (2.5 U/L, 0.3 μL), and total DNA (1 μL) using an Eppendorf 580BR Thermal Cycler. PCR cycling parameters for the six regions were as follows: a 94°C initial hot start for 3 min, followed by 32 cycles at 94°C for 30 s, 50°C for 30 s, 72°C for 60–70 s, and a final extension at 72°C for 7 min.

Amplified products were sequenced by the SinoGenoMax Co., LTD. (Beijing, China). Raw sequence fragments were assembled using Sequencher v.4.1.4 (Gene-Codes Corporation, Ann Arbor, Michigan, USA). Sequences were aligned using CLUSTAL X [30] with default settings and manual-adjusted following the similarity criterion [31] using Se–Al v.2.0 [32] to improve the alignment. Regions where homology was difficult to assess (the 78 bp in the *psbA-trnH* region) were excluded from the final dataset.

### Data analysis

An incongruence length difference (ILD) test was performed for topological incongruence among partitions [33]. Given that the plastid genome behaves as a single-linked region exhibiting low levels of variation, four plastid regions were combined as a priori. Congruence between ETS and ITS and combined cpDNA and nrDNA were examined using PAUP v4.0a150 [34]. Following Cunningham [35], no case of strongly supported incongruence was detected between ETS and ITS (P = 0.06), while it existed between cpDNA and nrDNA (P = 0.01). However, there was no significant conflict among highly supported subclades, and the combined data yielded a better tree than the individual ones, we combined the cpDNA and nrDNA for final analysis.

Phylogenetic analyses for chloroplast, nuclear, and combined datasets were performed using the Maximum Parsimony (MP) in PAUP*, Maximum Likelihood (ML) in RAxML [36], and Bayesian inference (BI) in Mrbayes 3.2 [37]. MP analyses were performed following our previous work [15]. ML analyses were conducted using RAxML v. 8.1.12 with 1000 replicates under the GTRGAMMA model as implemented on the HiPerGator 2.0 at the University of Florida. Prior to ML and BI analyses, a model of sequence evolution for each matrix was determined using Modeltest 3.7 [38] as implemented in MrMTgui [39] based on the Akaike information criterion [40]. For BI analyses, the Markov chain Monte Carlo (MCMC) algorithm was performed for each dataset, with three hot chains and one cold chain for 2×10^6^ generations in parallel mode. Trees were sampled every 1000 generations beginning with a random tree. The run was stopped when the average standard deviation of split frequencies was less than 0.005 in all the cases in this study. Bayesian posterior probabilities (BI_PP_) was calculated as the 50% majority rule consensus of all sampled trees with the first 20% discarded as burn-in. MP_BS_, ML_BS_ and BI_PP_ are presented on the phylogenetic trees.

## Results and Discussion

### Dataset characteristics

**Fig 1:**
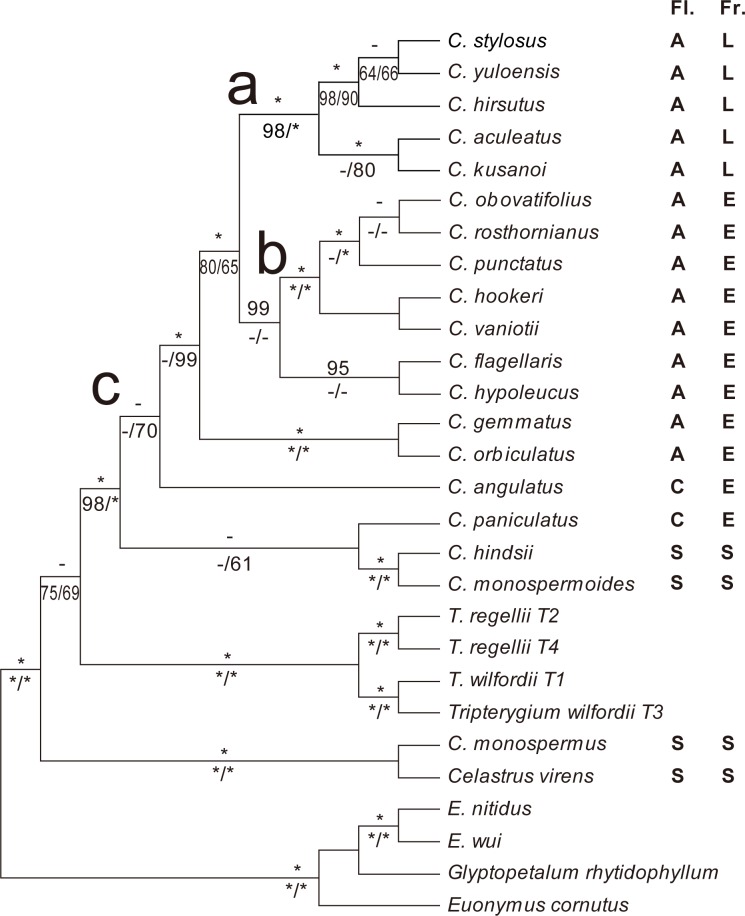
Bayesian inference of the phylogenetic relationship between Chinese *Celastrus* and *Tripterygium* from combined chloroplast and nuclear DNA datasets. Support values are presented above (BI_PP_, >95 is shown) and below (MP_BS_ and ML_BS_, >50 is shown) the branches (-: support value <95 for BI_PP_ or 50 for MP_BS_ and ML_BS_; *: full support value). Fl.: Subgeneric classification of Chinese *Celastrus* based on inflorescence, fruit and seed in *Flora Reipublicae Popularis Sinicae*, A: Sect. *Axillares*; C: Sect. *Celastrus*; S: Sect. *Sempervirentes*. Fr.: Subgeneric classification of Chinese *Celastrus* based on fruit and seed proposed in this study, L: Sect. *Lunatus*, E: Sect. *Ellipticus*, S: Sect. *Sempervirentes*.

Detailed information on both individual data partitions and the combined data matrix is summarized (Table 1). The result shows that tree topology and clade support value generated by six single markers is poor (Fig A-F in S1 File). Tree topology and clade support value of combined chloroplast markers (Fig G in S1 File) are better than combined nuclear markers (Fig H in S1 File). The combined chloroplast and nuclear markers obtained the best tree topology and clade support value. Considering that trees generated from MP, ML and BI were essentially the same, the 50% majority rule consensus tree obtained from BI of the combined ncDNA+cpDNA dataset was selected for final analysis (Fig 1).

**Table 1:** Statistics from phylogenetic analyses of individual and combined data sets.

	Nuclear markers			Plastid markers					Combined plastid and nuclear data
	ETS	ITS	Combined nuclear data	*psbA-trnH*	*rbcL*	*rpl16*	*trnL-F*	Combined plastid data	
Number of taxa	28	28	28	28	27	28	28	28	28
Aligned length	337	653	990	581	1061	1141	1086	3869	4859
Number of variable sites	151	208	359	120	45	235	122	522	881
Parsimony informative characters (%)	107 (31.75)	149 (22.82)	256 (25.86)	67 (11.53)	28 (2.64)	108 (9.47)	73 (6.72)	276 (7.13)	532 (10.95)
Tree length (steps)	289	411	711	181	66	327	137	724	1452
Consistency Index	0.70	0.65	0.66	0.76	0.73	0.82	0.96	0.80	0.72
Retention Index	0.81	0.78	0.78	0.83	0.81	0.81	0.97	0.84	0.80
Model selected by AIC*	HKY+G	GTR+I+G	GTR+I+G	GTR+I+G	TVM+I+G	K81uf+G	TVM+I	TVM+I+G	GTR+I+G

*AIC: Akaike information criterion.

Phylogenetic trees obtained from nuclear ETS and ITS are much better resolved (Fig A-B in S1 File), while trees of single chloroplast markers are generated with many polytomy and low clade support value (Fig C-F in S1 File). Trees of combined nuclear and chloroplast markers are better resolved than that of single markers, respectively (Fig G-H in S1 File). After combination of chloroplast and nuclear markers, the phylogenetic tree obtained the best tree topology and clade support value. Take into consideration the result of present and previous studies [15], it is implied that the combined data can provide better information for molecular identification in the genus *Celastrus*. Considering that several samples for each species are deposited in our laboratory, a standard DNA barcoding analysis is programmed.

### Phylogenetic reconstruction and medicinal implications

A comprehensive molecular analysis of Chinese *Celastrus* and *Tripterygium* was performed. The relationship between *Tripterygium* and *Celastrus* was further investigated and determined to be closer than that previously reported. Four *Tripterygium* samples collected from Northeast to Southwest China, grouped as two subclades, corroborated the results by Law et al. [21]. *Tripterygium* was nested in the base of the *Celastrus* tree with a stronger support value than our previous result [15]. Although the support value of BI was low (BI_pp_ = 91, not presented on the tree), a moderate MP_BS_ and ML_BS_ value were obtained (MP_BS_ = 75 (vs. 73 in previous study), ML_BS_ = 69 in this study; not calculated in the previous study), implying that the monophyletic group *Tripterygium* represent a natural clade in *Celastrus*. Paraffin section analysis of flower development between representatives of these two genera may provide additional information and verify their relationship; experiments addressing these issues are currently underway in our laboratory.

**Fig 2:**
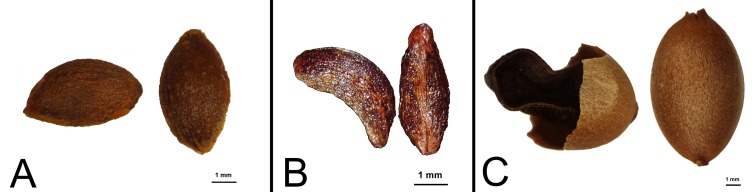
Seed type of *Celastrus* in China. 3-6-seeded fruit with ellipsoid seed (A, without aril), 3-6-seeded fruit with lunate seed (B, without aril) and 1-seeded fruit with ellipsoid seed (C, covered by dry aril).

Phylogenetic position of *C*. *yuloensis* is resolved in a monophyletic clade in the present study (Fig 1 clade a), which provides important evidence for subgeneric classification scheme in *Celastrus*. Although Sect. *Celastrus*, Sect. *Axillares* and Sect. *Sempervirentes* are divided for Chinese *Celastrus*, none of they are supported by molecular result [15]. However, a monophyletic group including *C*. *aculeatus*, *C*. *kusanoi*, *C*. *stylosus* and *C*. *hirsutus* was fully supported by both molecular result and morphological characteristics (cauline inflorescence and lunate seeds) in our previous work. Although morphologically similar to both *C*. *stylosus* and *C*. *hirsutus*, *C*. *yuloensis* differs from them with several characteristics, especially the long (vs. short) axillary (vs. cauline) cymes. In the present study, *C*. *yuloensis* is resolved as the most closet sister of the above two species and nested in the same monophyletic clade with approximately full support value (BI_pp_ = 100, MP_BS_ = 98, ML_BS_ = 90), which indicate that fruit and seed characteristics are better than inflorescence for subgeneric classification in *Celastrus*. There are three kinds of fruit and seed type in Chinese *Celastrus*: the first type contains species with 3-6-seeded fruit and ellipsoid seed ≤5 mm in length (Fig 2A), the second type includes species with 3-6-seeded fruit and lunate seed ≤6 mm in length (Fig 2B), and the third type is composed those of 1-seeded fruit and elliptic seed ≥8 mm in length (Fig 2C). Combing morphological characters and molecular phylogenetic results, a new morphology-based section for Chinese *Celastrus* is provided here: Sect. *Sempervirentes* CY Cheng & TC Kao, including 1-seeded fruit species such as *C*. *virens*, *C*. *monospermoides*, *C*. *monospermus* and *C*. *hindsii*; Sect. *Lunatus* XY Mu & ZX Zhang, **sect. nov.**, including 3-6-seeded fruit and lunate seed species such as *C*. *aculeatus*, *C*. *kusanoi*, *C*. *stylosus*, *C*. *hirsutus* and *C*. *yuloensis*; Sect. *Ellipticus* XY Mu & ZX Zhang, **sect. nov.**, including the remaining 11 3-6-seeded fruit and elliptic seed species.

**Fig 3:**
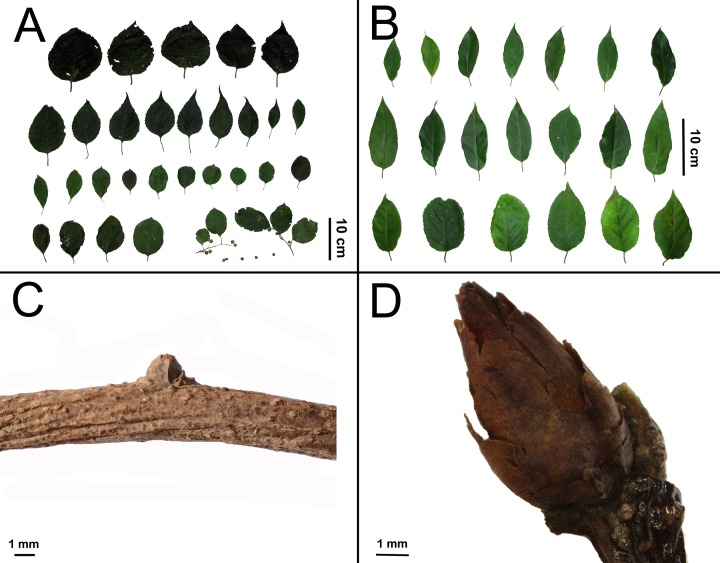
Leaf and bud morphology of *Celastrus orbiculatus* and *C*. *gemmatus*. A, C: Leaves and bud of *C*. *orbiculatus*; B, D: Leaves and bud of *C*. *gemmatus*.

The morphological complex consisting of five species was detected as a monophyletic group (clade b), whose inter-species relationships are still obscure, and need further clarification. *Celastrus orbiculatus*, a species frequently used in TCM, neighbors *C*. *gemmatus*. The former is morphologically similar to the latter, except its mostly orbicular (vs. elliptic) leaves (Fig 3A and 3B), and short and round buds < 4 mm (vs. elliptic buds >5 mm, Fig 3C and 3D). *Celastrus orbiculatus* is mostly distributed at north of Qinling Mountains while *C*. *gemmatus* is mainly distributed at south of it. However, it is very difficult to distinguish them in Qinling-Dabie Mountains. It is interesting and deserving to further investigate the evolutionary history and phylogeography of these two species, and the potential influence of Qinling-Dabie Mountains as vicariance in speciation. Based on comprehensive specimens examinations of *Celastrus* in main herbaria of China (e.g. CDBI, IBK, IBSC, KUN, PE, PEM, SZ and WH), specimens of other species are frequently misidentified as *C*. *orbiculatus*. Celastrol extracted from bark of *C*. *orbiculatus* is reported recently [41], while related plants are collected in the field from Yangchun City of Guangdong Province. However, Guangdong Province is located out of the distribution range of *C*. *orbiculatus*. *Celastrus angulatus*, positioned at foot of clade c, is one of the most famous TCMs in *Celastrus* that widely distributed in China covering subtropical to northern temperate areas, and can be recognized by its relatively large leaves and terminal long panicles. Life form is an obvious difference between clade c and the remaining *Celastrus* species. Plants of the former are deciduous and distributed in subtropical to temperate areas, whereas those of the latter are mostly evergreen and grow in tropical to subtropical regions. Although reported as deciduous, individuals of *C*. *paniculatus* are more similar to evergreens in that they also grow in subtropical and tropical zones.

### Phylogenetic-chemotaxonomic relationship

The inter-genus relationship concluded by molecular analysis was consistent to the chemical and pharmacological revision of *Celastrus*, *Euonymus* and *Tripgerygium*. Based on the latest chemical reviews [7–8], *Tripterygium* species share a number of common chemical constituents with *Celastrus* species including sesquiterpenes, diterpenes, triterpenes, alkaloids, and flavonoids. However, chlorogenic acid reported in *Euonymus* is not reported in these *Celastrus* and *Tripterygium* [42]. There are 13 dihydroagarofurans (sesquiterpene) in *Tripterygium*, whereas 93 were found mostly in *C*. *angulatus*, *C*. *orbiculatus* and *C*. *paniculatus*. Similarly, 6 euonymine-type sesquiterpene alkaloids were found in *Tripterygium*, and 4 were found in *C*. *hindsii* and *C*. *gemmatus*; friedooleananes with a benzenoid ring were only found in *C*. *paniculatus* and *Tripterygium*. According to the molecular phylogenetic tree, *C*. *hindsii*, *C*. *monospermus*, *C*. *monospermoides* and *C*. *virens* were very close to *Tripterygium* species. The consistency of chemotaxonomic and phylogenetic data indicated that some *Celastrus* species might have similar chemical components with *Tripterygium* species. Considering the prospects in medicinal use, more attention should be paid on these *Celastrus* species, especially *C*. *monospermus*, *C*. *monospermoides*, and *C*. *virens* due to their unique merits. First, these species are evergreen, are widely distributed in Southeast to Southwest Asia, and can provide more rural material. Second, trunks of these species grow faster than others, and can thus provide more material for medicinal studies and industrial production. Third, fruits of these species are three to five times larger than that of other species, and their maturation rates are high, thereby providing an increased amount of seeds and arils.

## Conclusion

The molecular analyses performed in this study provide a better resolved phylogenetic relationship of two genera of Chinese important medicinal plants, *Tripterygium* and *Celastrus*. *Tripterygium* was determined to be a monophyletic clade of *Celastrus* with a strong support value. The phylogenetic position of a unique species, *C*. *yuloensis*, is resolved in a monophyletic clade with approximately full support value, which implies that fruit and seed type are better characteristics for subgeneric classification of Chinese *Celastrus* than inflorescence. A new section scheme combined morphological and molecular result is proposed for Chinese *Celastrus*. A more comprehensive analysis with additional molecular markers will be helpful to differentiate and elucidate the phylogenetic relationships of the morphological complex. The result of combined nuclear and chloroplast DNA provides reference for molecular identification of Chinese *Celastrus* and *Tripterygium* species. Furthermore, evergreen species of *Celastrus* distributed in Southeast China, such as *C*. *monospermus*, *C*. *monospermoides*, and *C*. *virens*, are highly recommended as potentially important medicinal germplasm in TCM for several merits.

## Supporting Information

S1 File Tree topology and clade support value of single and combined markers in this study (A-H Figures). (DOCX)

S1 Table Voucher information (samples and OUT, voucher information, locality) and GenBank accession numbers used in this study. (DOCX)
