# Supplementary material for: Molecular Analysis of Chinese Celastrus and Tripterygium and Implications in Medicinal and Pharmacological Studies
Source: PLoS One. 2017 Jan 12;12(1):e0169973. doi: 10.1371/journal.pone.0169973 (PMC5231332; doi:10.1371/journal.pone.0169973)
Supplement: S1 File — (DOCX) [file pone.0169973.s001.docx]

Fig A. Bayesian inference of the phylogenetic relationship between Chinese *Celastrus* and *Tripterygium* from nuclear ETS datasets. Support values are presented above (BI_PP_, >95 is shown) and below (MP_BS_ and ML_BS_, >50 is shown) the branches (-: support value <95 for BI_PP_ or 50 for MP_BS_ and ML_BS_; *: full support value).

Fig B. Bayesian inference of the phylogenetic relationship between Chinese *Celastrus* and *Tripterygium* from nuclear ITS datasets. Support values are presented above (BI_PP_, >95 is shown) and below (MP_BS_ and ML_BS_, >50 is shown) the branches (-: support value <95 for BI_PP_ or 50 for MP_BS_ and ML_BS_; *: full support value).

Fig C. Bayesian inference of the phylogenetic relationship between Chinese *Celastrus* and *Tripterygium* from chloroplast *psbA-trnH* datasets. Support values are presented above (BI_PP_, >95 is shown) and below (MP_BS_ and ML_BS_, >50 is shown) the branches (-: support value <95 for BI_PP_ or 50 for MP_BS_ and ML_BS_; *: full support value).

Fig D. Bayesian inference of the phylogenetic relationship between Chinese *Celastrus* and *Tripterygium* from chloroplast *rbcL* datasets. Support values are presented above (BI_PP_, >95 is shown) and below (MP_BS_ and ML_BS_, >50 is shown) the branches (-: support value <95 for BI_PP_ or 50 for MP_BS_ and ML_BS_; *: full support value).

Fig E. Bayesian inference of the phylogenetic relationship between Chinese *Celastrus* and *Tripterygium* from chloroplast *rpl16* datasets. Support values are presented above (BI_PP_, >95 is shown) and below (MP_BS_ and ML_BS_, >50 is shown) the branches (-: support value <95 for BI_PP_ or 50 for MP_BS_ and ML_BS_; *: full support value).

Fig F. Bayesian inference of the phylogenetic relationship between Chinese *Celastrus* and *Tripterygium* from chloroplast *trnL-F* datasets. Support values are presented above (BI_PP_, >95 is shown) and below (MP_BS_ and ML_BS_, >50 is shown) the branches (-: support value <95 for BI_PP_ or 50 for MP_BS_ and ML_BS_; *: full support value).


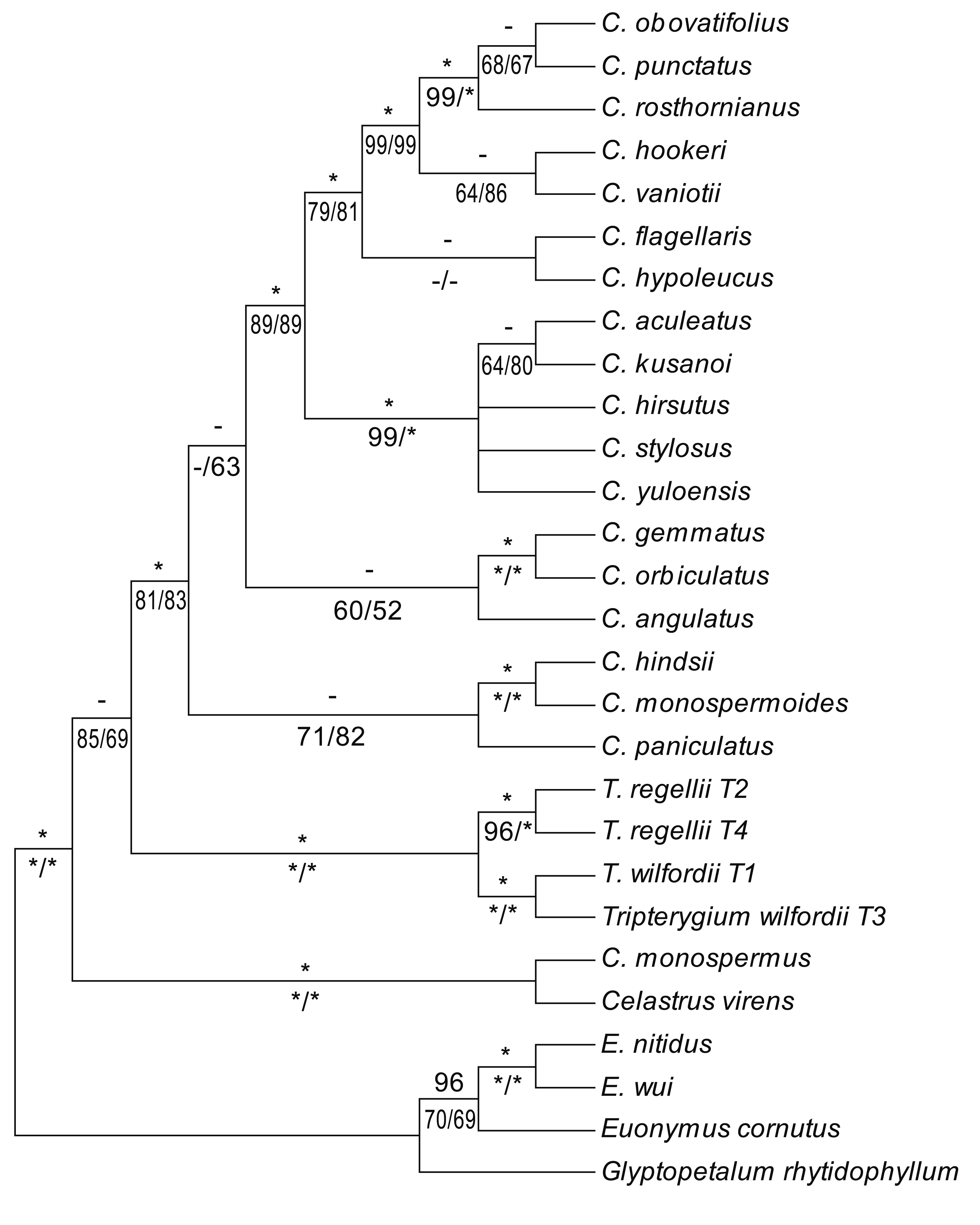


Fig G. Bayesian inference of the phylogenetic relationship between Chinese *Celastrus* and *Tripterygium* from combined chloroplast DNA datasets. Support values are presented above (BI_PP_, >95 is shown) and below (MP_BS_ and ML_BS_, >50 is shown) the branches (-: support value <95 for BI_PP_ or 50 for MP_BS_ and ML_BS_; *: full support value).


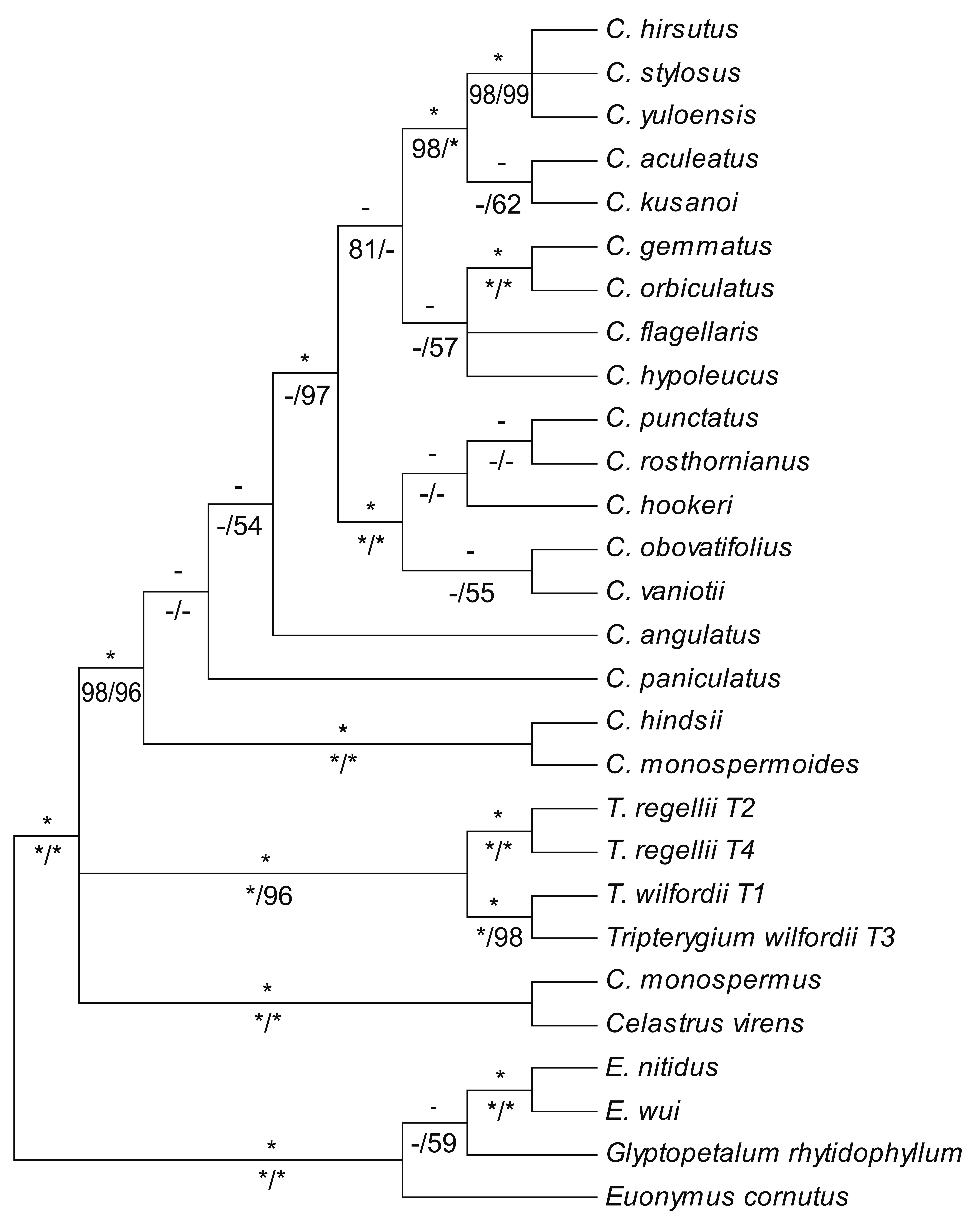


Fig H. Bayesian inference of the phylogenetic relationship between Chinese *Celastrus* and *Tripterygium* from combined nuclear DNA datasets. Support values are presented above (BI_PP_, >95 is shown) and below (MP_BS_ and ML_BS_, >50 is shown) the branches (-: support value <95 for BI_PP_ or 50 for MP_BS_ and ML_BS_; *: full support value).
