# Supplementary material for: Molecular Analysis of Chinese Celastrus and Tripterygium and Implications in Medicinal and Pharmacological Studies
Source: PLoS One. 2017 Jan 12;12(1):e0169973. doi: 10.1371/journal.pone.0169973 (PMC5231332; doi:10.1371/journal.pone.0169973)
Supplement: S1 Table — (DOCX) [file pone.0169973.s002.docx]

Supplementary Table 1. Voucher information (samples and OUT, voucher information, locality) and GenBank accession numbers (–: not sequenced) used in this study. All specimens are deposited in BJFC except special instruction.

| Taxa and number | Location | Latitude | Longitude | Altitude (m) | GenBank number | | | | | |
| --- | --- | --- | --- | --- | --- | --- | --- | --- | --- | --- |
|  |  |  |  |  | ETS | ITS | *psbA-trnH* | *rpl16* | *trnL-F* | *rbcL* |
| *Celastrus angulatus* Maxim. | Shennongjia, Hubei Province, China | N31°22′50.44″ | E110°29′16.53″ | 804 | JQ424040 | JQ424097 | JQ424151 | JQ424207 | JQ424261 | KT258933 |
| *Celastrus flagellaris* Rupr. | Jilin, Jilin Province, China | N43°43′38.00″ | E126°42′44.20″ | 663 | JQ424044 | JQ424101 | JQ424154 | JQ424211 | JQ424265 | KT258934 |
| *Celastrus gemmatus* Loes. | Shennongjia, Hubei Province, China | N31°22′50.44″ | E110°29′16.53″ | 804 | JQ424045 | JQ424102 | JQ424155 | JQ424212 | JQ424266 | KT258935 |
| *Celastrus glaucophyllys* Rehd & Wils. | Nielamu, Xizang Autonomous Region, China | N27°57′50.70″ | E85°58′11.40″ | 2431 | KT258912 | KT258919 | KT258926 | KT258960 | KT258967 | KT258936 |
| *Celastrus hindsii* Benth. | Xiamen, Fujian Province, China | N24°26′37.78″ | E118°6′5.90″ | 128 | KT258913 | KT258920 | KT258927 | KT258961 | KT258968 | KT258937 |
| *Celastrus hirsutus* Comb. | Wenshan, Yunnan Province, China | N23°28′56.60″ | E103°56′32.26″ | 1510 | JQ424051 | JQ424108 | JQ424161 | JQ424218 | JQ424272 | KT258938 |
| *Celastrus hypoleucus* (Oliv.) Warb. ex Loes. | Luanchuan, Henan Province, China | N33°45′56.40″ | E111°38′37.08″ | 813 | JQ424055 | JQ424112 | JQ424165 | JQ424222 | JQ424276 | KT258939 |
| *Celastrus kusanoi* Hayata | Jiayi, Taiwan Province, China | N23°31′7.20″ | E120°46′49.78″ | 1395 | KT258915 | KT258922 | KT258929 | KT258963 | KT258970 | – |
| *Celastrus monospermoides* Loes. | Xishuangbanna, Yunnan Province, China | N21°36′26.99″ | E101°34′58.82″ | 687 | JQ424060 | JQ424116 | JQ424170 | JQ424227 | JQ424281 | KT258940 |
| *Celastrus monospermus* Roxb. | Xishuangbanna, Yunnan Province, China | N21°36′26.99″ | E101°34′58.82″ | 687 | JQ424062 | JQ424117 | JQ424172 | JQ424229 | JQ424283 | KT258941 |
| *Celastrus oblanceifolius* Wang & Tsoong | Wuyi Mountain, Fujian Province, China | N27°39′14.04″ | E117°56′7.21″ | 228 | JQ424064 | JQ424119 | JQ424174 | JQ424231 | JQ424285 | KT258942 |
| *Celastrus obovatifolius* Mu & Zhang | Luanchuan, Henan Province, China | N33°45′56.40″ | E111°38′37.08″ | 813 | JQ424065 | JQ424120 | JQ424175 | JQ424232 | JQ424286 | KT258943 |
| *Celastrus orbiculatus* Thunb. | Beijing, China | N39°59′18.14″ | E116°12′32.32″ | 76 | JQ424067 | JQ424122 | JQ424177 | JQ424234 | JQ424288 | KT258944 |
| *Celastrus paniculatus* Willd. | Ledong, Hainan Province, China | N18°44′19.26″ | E108°58′9.01″ | 294 | KT258914 | KT258921 | KT258928 | KT258962 | KT258969 | KT258945 |
| *Celastrus rosthornianus* Loes. | Shennongjia, Hubei Province, China | N31°22′50.44″ | E110°29′16.53″ | 804 | JQ424076 | JQ424131 | JQ424186 | JQ424242 | JQ424297 | KT258947 |
| *Celastrus punctatus* Thunb. | Jiayi, Taiwan Province, China | N23°31′7.20″ | E120°46′49.78″ | 1395 | JQ424073 | JQ424128 | JQ424183 | JQ424239 | JQ424294 | KT258946 |
| *Celastrus stylosus* Wall. | Tengchong, Yunnan Province, China | N25°13′18.46″ | E98°36′53.90″ | 1633 | JQ424080 | JQ424135 | JQ424190 | JQ424246 | JQ424301 | KT258948 |
| *Celastrus yuloensis* Mu | Xishuangbanna, Yunnan Province, China | N21°59′22.85″ | E101°5′20.12″ | 1255 | KT258916 | KT258923 | KT258930 | KT258964 | KT258971 | KT258951 |
| *Celastrus vaniotii* (Levl.) Rehd. | Emeishan, Sichuan Province, China | N29°32′46.55″ | E103°18′41.14″ | 2337 | JQ424084 | JQ424139 | JQ424194 | JQ424250 | JQ424305 | KT258949 |
| *Celastrus virens* (Wang & Tang) Cheng & Kao | Xishuangbanna, Yunnan Province, China | N21°36′26.99″ | E101°34′58.82″ | 687 | JQ424086 | JQ424141 | JQ424196 | JQ424251 | JQ424307 | KT258950 |
| *Tripterygium regelii* Sprague et Takeda T2 | Jilin, Jilin Province, China | N43°43′38.00″ | E126°42′44.20″ | 663 | JQ424093 | JQ424147 | JQ424203 | JQ424257 | JQ424314 | KT258956 |
| *Tripterygium regelii* Sprague et Takeda T4 | Antu, Jilin Province, China | N42°27′51.71″ | E128°8′22.29″ | 676 | KT258917 | KT258924 | KT258931 | KT258965 | KT258972 | KT258957 |
| *Tripterygium wilfordii* Hook. f T1 | Kunming, Yunnan Province, China | N25°8′32.91″ | E102°44′52.29″ | 1982 | JQ424094 | JQ424148 | JQ424204 | JQ424258 | JQ424315 | KT258958 |
| *Tripterygium wilfordii* Hook. f T3 | Leishan, Guizhou Province, China | N26°22′56.12″ | E108°6′22.45″ | 1081 | KT258918 | KT258925 | KT258932 | KT258966 | KT258973 | KT258959 |
| *Euonymus cornutus* Hemsl. | Luanchuan, Henan Province, China | N33°45′56.40″ | E111°38′37.08″ | 813 | JQ424089 | JQ424143 | JQ424199 | JQ424253 | JQ424310 | KT258952 |
| *Euonymus nitidus* Benth. | Fangchenggang, Guangxi Autonomous Region, China | N21°52′19.45″ | E108°7′27.36″ | 81 | JQ424090 | JQ424144 | JQ424200 | JQ424254 | JQ424311 | KT258953 |
| *Euonymus wui* Ma | Longzhou, Guangxi Autonomous Region, China | N22°26′44.70″ | E107°6′1.57″ | 152 | JQ424091 | JQ424145 | JQ424201 | JQ424255 | JQ424312 | KT258954 |
| *Glyptopetalum rhytidophyllum* (Chun & How) Cheng | Longzhou, Guangxi Autonomous Region, China | N22°26′44.70″ | E107°6′1.57″ | 152 | JQ424094 | JQ424148 | JQ424204 | JQ424258 | JQ424315 | KT258955 |
